# *In Vitro* Toxicological Assessment of Cylindrospermopsin: A Review

**DOI:** 10.3390/toxins9120402

**Published:** 2017-12-16

**Authors:** Silvia Pichardo, Ana M. Cameán, Angeles Jos

**Affiliations:** Area of Toxicology, Faculty of Pharmacy, University of Sevilla, C/Profesor García González 2, 41012 Sevilla, Spain; spichardo@us.es (S.P.); angelesjos@us.es (A.J.)

**Keywords:** cylindrospermopsin, *in vitro*, cytotoxicity, oxidative stress, genotoxicity

## Abstract

Cylindrospermopsin (CYN) is a cyanobacterial toxin that is gaining importance, owing to its increasing expansion worldwide and the increased frequency of its blooms. CYN mainly targets the liver, but also involves other organs. Various mechanisms have been associated with its toxicity, such as protein synthesis inhibition, oxidative stress, etc. However, its toxic effects are not yet fully elucidated and additional data for hazard characterization purposes are required. In this regard, *in vitro* methods can play an important role, owing to their advantages in comparison to *in vivo* trials. The aim of this work was to compile and evaluate the *in vitro* data dealing with CYN available in the scientific literature, focusing on its toxicokinetics and its main toxicity mechanisms. This analysis would be useful to identify research needs and data gaps in order to complete knowledge about the toxicity profile of CYN. For example, it has been shown that research on various aspects, such as new emerging toxicity effects, the toxicity of analogs, or the potential interaction of CYN with other cyanotoxins, among others, is still very scarce. New *in vitro* studies are therefore welcome.

## 1. Introduction

The cyanobacterial toxin Cylindrospermopsin (CYN) is a tricyclic alkaloid that consists of a tricyclic guanidine moiety combined with hydroxymethyluracil [1]. Owing to its zwitterionic nature, CYN is a highly water-soluble compound [2]. Currently, five analogs of CYN are known, namely CYN, 7-epi-CYN, 7-deoxy-CYN, and the two recently characterized congeners, 7-deoxydesulfo-CYN and 7-deoxydesulfo-12-acetyl-CYN [3,4].

It was first reported in 1979 after a hepatoenteritis outbreak occurred in Palm Island, northern Queensland, Australia [5], owing to a *Cylindrospermopsis raciborskii* bloom in the local drinking water supply. Nowadays, the variety of identified CYN-producing cyanobacteria species has increased considerably (i.e., *Umezakia natans*, *Aphanizomenon ovalisporum*, *Raphidiopsis curvata*, *Anabaena bergii*, *Aphanizomenon flos-aquae*, *Anabaena lapponica*, etc.). *Aphanizomenon gracile* and *A. flos-aquae* are the most important CYN producers in Europe [6]. Moreover, for most of the known CYN-producing species, both CYN-producing and nonproducing strains have been observed [4].

The occurrence of CYN and/or CYN-producing species has been reported worldwide, in Germany, Saudi Arabia, Australia, China, Israel, Spain, United States of America (USA), Italy, Finland, Poland, Portugal, France, etc. [4,7]. The ever-expanding distribution of CYN producers into temperate zones is heightening concern that this toxin will represent serious human, as well as environmental, health risks across many countries [8]. Among the reasons for the increase in extension and frequency of their blooms, i.e., cyanobacterial growth at high densities, are anthropogenic activities and climate changes.

With regard to environmental concentrations of CYN, it is usually found in the range of 1–10 µg/L [9], the highest values reported being 589 µg/L in an aquaculture pond in Queensland [10] and 800 µg/L in a farm dam in Australia [11,12].

CYN can adversely affect both humans and the environment. Human exposure to CYN may occur by different pathways. Dermal contact with CYN may occur during showering or bathing, or during recreational activities such as wading, swimming, boating, or water skiing. Also, by ingesting toxin-contaminated water during recreational activities or by the ingestion of food or water contaminated with the toxin. In fact, it has been demonstrated that cyanobacterial toxins (including CYN) are able to accumulate in edible plants [13,14], fish [15], crustaceans [10], etc., an aspect that has been reviewed by Gutiérrez-Praena et al. [16]. To protect consumers from the adverse effects of CYN, a provisional Tolerable Daily Intake (TDI) of 0.03 µg/kg body weight (b.w.) has been established [17]. Moreover, these authors also proposed a guideline value of 1 µg/L for CYN in drinking water.

CYN appears to be a molecule with a wide range of toxic effects. The toxin primarily targets the liver, but it is also a general cytotoxin that attacks the eye, spleen, kidney, lungs, thymus, heart, etc. [18]. The lack of a specific target for CYN hinders further efforts to understand its potent toxicity and to define acceptable thresholds of exposure [4]. In this line, the European Authority of Food Safety (EFSA) considers that there are also data gaps regarding the characterization of the toxicological profile of cyanotoxins other than microcystins [19].

If we focus on the toxicological evaluation that is required for hazard characterization purposes, *in vitro* methods play an important role. The use of *in vitro* model systems in toxicity testing has many advantages, including a decrease in animal numbers, a reduced cost of animal maintenance and care, a small quantity of chemicals needed for testing, shortening of the time needed, and an increase in throughput for evaluating multiple chemicals and their metabolites [20]. *In vitro* systems can also be used to study chemical metabolism, evaluate toxicity mechanisms, measure enzyme kinetics, and examine dose-response relationships [20,21]. Thus, the aim of this work was to compile and evaluate the *in vitro* data dealing with CYN that are available in the scientific literature, focusing on its toxicokinetics and its main toxicity mechanisms. This analysis would be useful to identify research needs and data gaps in order to complete knowledge about the toxicity profile of CYN.

## 2. Basal Cytotoxicity Assays and Morphological Studies

Table 1 and Table 2 show the various CYN *in vitro* studies that are dealing with these two basic toxicological features, respectively [9,22,23,24,25,26,27,28,29,30,31,32,33,34,35,36,37,38,39,40,41,42]. *In vivo* studies in mice suggest that liver is a major target organ; in fact, CYN has traditionally been classified as a hepatotoxin [43]. Consequently, most of the first *in vitro* studies performed to investigate the cytotoxicity of this cyanotoxin used primary rodent hepatocytes [44]. Primary rat hepatocytes exposed for 18 h to 3.3–5 µM of CYN isolated from *C. raciborskii* cultures resulted in significant cell death [22]. Subsequently, the same authors studied the toxicity of natural and synthetic CYN and its analogs in rat hepatocytes in order to investigate the role of various chemical groups [24]. They showed that the sulfate group and the orientation of the hydroxyl group at C-7 were not relevant in CYN biological activity. Recently, the toxicity of four CYN analogs, which are differing in the length of tether guanidine and uracil groups, and the presence or absence of a hydroxyl group, was studied. Preliminary findings revealed that the −OH group at C-7 of the toxin was responsible of toxic effects induced on human neutrophils [9]. In addition, Neumann et al. [26] compared the toxicity of CYN and its analog deoxycylindrospermopsin (deoxy-CYN), showing similar effects on cell viability and proliferation in different cell lines.

Given that metabolism plays an important role in the toxicity of CYN [24,28,45], the influence of metabolic inhibitors has also been studied. Co-exposure of CYN and CYP inhibitors on primary mouse hepatocytes for 18 h demonstrated the effectiveness of ketoconazole and SKF525A in decreasing CYN toxicity, while furafylline and omeprazole showed a moderate protective effect [26]. Similarly, various isoforms of CYP have been induced by ethanol, rifampicin, and phenobarbital, in order to assess the influence of each CYP on CYN biotransformation and further toxicity [41]. The authors showed that CYP-induced HepG2 cells were more sensitive to CYN exposure with regard to the decrease of cell viability after 24 h of exposure to 10 µg/L CYN.

In addition, the toxicity of CYN on primary hepatocytes and permanent cell lines has been compared. After 72 h of exposure to pure standard CYN, the viability of KB cells decreased from 200 ng/mL, whereas the effective concentration was around 10-fold lower (25 ng/mL) in rat hepatocytes [23]. The higher sensitivity of isolated hepatocytes to CYN in comparison to permanent cell lines will be discussed further. However, CYN causes severe toxicity in a wide range of human cell lines from various target organs, such as liver, kidney, and intestine [23,27,31,32]. After rodent hepatocytes, the most used permanent cell lines have been Caco-2 cells (from human intestinal carcinoma) and HepG2 cells (derived from human hepatoma). The hepatic-derived cells have proved to be more sensitive than intestinal ones, while colon-derived cells are even less sensitive than the others [31]. This finding may be related to the limited CYN uptake in colon cells that is reported by several authors [39,46].

Most of the experiments that have demonstrated toxic effects have used 24 and 48 h of exposure. In fact, Young et al. [30] showed no cytotoxic effects in human granulosa cells that were exposed for 6 h to 1 µg/mL, although at longer exposure times (24–72 h) cell viability decreased. In contrast, short-term exposure of hepatic cells (C3A) to CYN (1–6 h) was shown to induce cytotoxicity at 24 h despite a washout and recovery incubation, demonstrating the apparently irreversible nature of CYN toxicity [32]. Apart from the time-dependent cytotoxicity, the toxic effects of CYN also increase with concentration [26,28,30,34,36,37,38,39,40]. Surprisingly, CYN decreased cell viability in fish hepatocytes that were exposed to the lowest concentrations (0.1 and 1 µg/L), but no significant effect was recorded in the exposure to the highest concentration assayed (10 µg/L) [41]. This is the only work performed on primary fish hepatocytes, so the unexpected behavior cannot be compared with similar experiments. The only report available carried out on fish cells did not use isolated hepatocytes and instead used the permanent fish cell line, PLHC-1, a hepatocellular carcinoma of the cyprinid fish *Poeciliopsis lucida* [34]. However, this study revealed time- and concentration-dependent cytotoxic effects, but at higher concentrations than in the above-mentioned fish hepatocytes (0.3–40 µg/mL). When considering that most of the studies have been performed on mammalian cells, more research is needed using *in vitro* experimental models of aquatic origin, because they may easily be exposed to CYN.

Cell death was also determined using the Annexin V kit in rat hepatocytes that were exposed to CYN [38]. After 6 h of exposure, cells were stained with Annexin V, indicating that apoptosis is rapidly induced by CYN. Only at the highest concentrations assayed (180 and 360 nM CYN) did the cells suffer loss of membrane integrity after 48 h, demonstrated by propidium iodide staining. In order to determine whether necrosis was also induced by CYN, the LDH released to the medium was determined in hepatocyte cultures treated with the toxin, showing positive results after 72 h of treatment. However, CHO-K1 cells that were exposed to CYN for 3 and 16 h did not result in a statistically significant enhancement of the frequency of early apoptotic cells [28]. Only at a longer incubation time (21 h) was a dose-dependent increase in the frequency of early apoptotic cells observed, which was significant at 1 and 2 μg/mL CYN. Similar results were observed in necrotic cells, which increased steadily after 21 h of exposure. Poniedziałek et al. [40] also reported that 1 µg/mL CYN was able to cause both apoptosis and necrosis in human lymphocytes after 6 h of exposure, although at 72 h only necrotic cells were found. In general, cell death may occur by apoptosis or necrosis, depending on physiological conditions, developmental stages, cell type, and nature of the death signal [33]. These authors reported 19% of apoptotic cells and 9% of necrosis after incubation with 10 µM CYN for 18 h. At longer exposure times (24 h, 48 h) in the presence of CYN, apoptosis became a necrotic process, attaining about 75% after 48 h of incubation, with 10 µM CYN.

Although morphological studies are very scarce, they are of great interest because they are more sensitive than cytotoxicity studies and can be used as an early indicator of damage that is induced in cells [36]. In this context, using microscopy, Gutiérrez-Praena et al. [37] observed apoptosis in human endothelium cells (HUVEC), which showed pleomorphic nuclei after being exposed to 0.375 µg/mL CYN. In addition, they also reported nucleolar segregation with altered nuclei, degenerated Golgi apparatus, and increases of granules. These authors found lipid degeneration, mitochondrial damage, and nucleolar segregation with altered nuclei in Caco-2 cells after exposure to 2.5 μg/mL CYN [36]. Also, Maire et al. [42] showed nuclear alteration in Syrian hamster embryo (SHE) cells after seven days of exposure to 10^−7^–10^−2^ ng/mL CYN. In the case of hepatic cells, no remarkable morphological changes were observed after 24 h incubation of Clone-9 cells with 5 mM CYN. However, after 48 h of exposure to the toxin, the cells showed evident disturbance, with signs of damage and detachment from the substrate [39]. Finally, significant morphological changes were also observed in various cell lines, BE2, MNA, and HepG2 cells, after exposure to 2.5 μg/mL CYN and deoxy-CYN. Moreover, BE2 and MNA cells underwent morphological changes that were indicative of apoptosis, such as cell shrinkage and cell rounding [29].

## 3. Toxicokinetic Studies

*In vitro* experiments provide a means of selectively measuring and estimating absorption, distribution, metabolism, and excretion (ADME) parameters [47]. Relevant studies on this topic are compiled in Table 3 [23,32,39,41,46,48]. The results that are obtained in these *in vitro* assays, once they have been properly analyzed, can be extrapolated reliably to the *in vivo* situation [47,49]. However, these data should also be completed with those that are obtained in physiologically-based toxicokinetic (PBTK) models for a better approach in the study of the toxicity of chemicals [50]. Such combinatorial approaches are very promising for the investigation of interspecies and intraspecies differences [51]. However, very few studies have been performed so far to study the ADME of CYN; and, they have focused mainly on absorption and metabolism.

The exact uptake mechanism of CYN has not been fully elucidated yet. The chemical characteristics of CYN, its size (415 Da), and hydrophilic nature indicate that it would be unlikely to cross the lipid bilayer of cell membrane, and therefore would need to be transported across the cell membrane [24,32]. Transport inhibitors have been used in order to clarify the CYN uptake mechanism that is involved [23]. Incubation with bile acids, cholate, and taurocholate, resulted in limited CYN uptake. A protective effect of bile acids was observed only after 48 h, but not at 72 h. These results showed that, although the bile acid transport system may participate in CYN uptake, another mechanism could be involved. In this context, a facilitated transport mechanism and active transport have been studied. Competition experiments excluded the uracil nucleobase transporter system as a potential mechanism for CYN uptake in Vero-GFP cells [32]. In addition, these authors confirmed that the uptake process is not energy-dependent because CYN entry also occurred at 4 °C, and at this temperature the energy-dependent cell processes are minimized. Similarly, no significant changes in CYN uptake were reported at 4 °C in comparison to 37 °C in Caco-2 intestinal cells [46]. However, a significant reduction in CYN transport was observed in the secretory direction when the temperature was decreased. Moreover, the main pathway that is involved in CYN uptake in intestinal cells was the paracellular route. As has been suggested with regard to other cell lines, a minor carrier-mediated transcellular transport has been indicated as a possible CYN uptake mechanism. This transport through the intestinal monolayer may be H^+^ and GSH-dependent, and energy and Na^+^-independent [46]. However, apart from these insights, intestinal uptake of CYN has not been reported, and it seems clear that intestinal absorption of CYN through Caco-2 cells is very limited. Fernandez et al. [39] reported that the passage of CYN across the intestinal monolayer was about 2.5% after 3 h and up to 20.5% after 24 h. Similarly, the permeability coefficients found in Pichardo et al. correlate well with very low *in vivo* absorption (below 20%) [46].

As mentioned earlier, differences in the toxicity exerted by CYN have been observed in a variety of experimental models [52]. These differences could be related to a highly active transport process in primary hepatocytes or other primary cells that may be absent in immortalized cell lines [39]. However, the grade of metabolic competency of each *in vitro* experimental model may also be of concern. In this context, the influence of CYN metabolism is an important key to understanding the toxicity that is exerted by CYN. In fact, it has been proposed that the higher sensitivity of hepatocytes exposed to CYN is due to bioactivation-dependent events [39,45,53]. In this regard, the activity of the cytochrome P450 (CYP450) enzyme system has been shown to be important for the development of CYN toxicity in hepatocyte cultures [22,53]. This finding could be the explanation for the lower toxicity observed in permanent cell lines, such as KB cells [23], HeLa cell types [11], and CHO-K1 cells [28], in comparison to primary rat hepatocytes, suggesting that CYP450 activity is higher in hepatocytes. Moreover, some authors have reported that the metabolic activation of CYN intensified the cytotoxic effect, indicating that S9 fraction-induced metabolism of CYN is important for its cytotoxic activity [22,28,45], and also in genotoxicity effects (see Section 4.3). Despite the use of broad-spectrum CYP inhibitors, the isoforms that are involved have not been identified so far [41]. However, other authors have reported that preinduction of expression of xenobiotic metabolism enzymes, such as CYPs, does not increase the toxicity of CYN [41]. Similarly, no evidence was found for phase I metabolites of CYN when studying metabolic conversion using HepaRG cells and different liver tissue fractions, so this metabolic activation plays only a minor role for CYN toxicity [48]. With regard to the other two toxicokinetic phases, no *in vitro* experiment has been carried out to study the distribution or excretion of CYN as far as we know, and *in vivo* studies are scarce [54].

## 4. Toxicity Mechanisms

### 4.1. Protein Synthesis Inhibition

The first evidence that CYN induced an irreversible protein synthesis inhibition *in vivo* in mice was reported by Terao et al. [55]. Moreover, they verified this finding and also found inhibitory effects of the toxin on globin synthesis in a rabbit reticulocyte cell-free system. Subsequently, Froscio et al. [45] also confirmed this effect on primary mouse hepatocytes. These authors stated that protein synthesis inhibition was a sensitive early indicator of cellular responses to CYN. Moreover, the inhibition of CYP450 activity diminished the toxicity of CYN, but not the effects on protein synthesis. This suggests that the parent compound and the possibly formed metabolites could exert toxicity with a different mechanism, also depending on CYN concentrations [7].

### 4.2. Oxidative Stress

Oxidative stress is one of the toxic mechanisms postulated as being responsible for CYN toxicity. The term oxidative stress has been defined as a serious imbalance between reactive oxygen species (ROS) production and antioxidant defenses [56]. Sies [57] defined it as “a disturbance in the pro-oxidant–antioxidant balance in favour of the former, leading to potential damage”. Other authors suggest that oxidative stress may be better defined as a disruption of redox signaling and control [58]. Table 4 [9,22,24,26,34,35,36,37,38,39,41,59,60,61,62,63] shows the *in vitro* studies available in the scientific literature dealing with oxidative stress induced by CYN.

Glutathione (GSH) is one of the major endogenous antioxidants that is produced by cells, so a deficit of it can play an important role in the potential induction of oxidative damage by xenobiotics. It is well known that CYN inhibits GSH synthesis. This statement derives from the studies performed by Runnegar et al. [22,24,59], who found that CYN caused a significant GSH fall in rat primary hepatocytes, and that potentiating effects were observed when cells were exposed concomitantly to CYN and a GSH inhibitor (propargylglycine) [22]. Moreover, they also addressed whether the fall in GSH was due to decreased GSH synthesis or increased GSH consumption, and they found that the inhibition of GSH synthesis was the predominant mechanism for the CYN-induced fall in GSH [59]. The GSH depletion was related to the cytotoxicity observed.

The effect of CYN on GSH content is one of the oxidative stress biomarkers that has been most extensively studied in the scientific literature. Apart from Runnegar et al. [22,24,59], there are other authors who also found a depletion (i.e., Humpage et al. [26]), but in other cases, different results have been reported. Thus, Liebel et al. [35] did not find changes in this parameter in *Prochilodus lineatus* primary hepatocytes, while other authors showed a significant increase. Gutiérrez-Praena et al. [34] observed a dual response in the fish PLHC-1 cell line, with a significant increase at the lowest CYN concentration assayed (2 µg/mL) and a significant reduction at the highest one (8 µg/mL). In human HUVEC cells, on the other hand, they found a concentration-dependent increase (from 0.375 to 1.5 µg/mL CYN) [37], and in human intestinal cells, the increase was only evident at the highest concentration tested (2.5 µg/mL) [36]. Other authors who found a significant increase were Fernández et al. [39], in the rat hepatic cell line Clone 9, and Silva et al. [63], in *Hoplias malabaricus* hepatocytes. In any case, it has been suggested that the GSH reduction does not contribute significantly to CYN acute toxicity *in vivo* [64].

Various studies have also investigated the effect of CYN on Gamma Glutamylcysteine Synthetase (GCS) activity, as this is the limiting enzyme in GSH synthesis. Runnegar et al. [59] concluded that CYN inhibits GSH synthesis, and this statement was based on the finding that an excess of a GSH precursor (*N*-acetylcysteine), which supported GSH synthesis in control cells, did not prevent the fall in GSH or toxicity that was induced by CYN. Other authors, however, observed a different response pattern. Thus, Gutiérrez-Praena et al. [34] found a significant increase at the lowest concentration that was assayed and a significant reduction at the highest one (8 µg/mL) in PLHC-1 cells. In human cell lines, on the other hand, only significant increases were observed at 2.5 µg/mL CYN in Caco-2 cells [36] and at 0.75–1.5 µg/mL in HUVEC cells [37]. Fernández et al. [39] also found a time-dependent increase of GCS levels in the rat hepatic cell line (Clone 9) that was exposed to 5 µM CYN.

A GSH depletion could be directly correlated, among other responses, with an increase in ROS levels. In this regard, it is remarkable that, in all of the reports selected, CYN exposure induced an enhancement of ROS content. This may play an important role in other toxic mechanisms, for instance, genotoxicity [26]. Other important oxidative biomarkers, however, such as lipid peroxidation, have scarcely been investigated, and different results have been obtained. Humpage et al. [26] observed no remarkable effects in primary mice hepatocytes, while increases were reported by Poniedziałek et al. [62] in human lymphocytes, and by Liebel et al. [35] in fish primary hepatocytes. These authors also found that CYN produced a variable effect in HepG2 cells [41]. It decreased LPO in cells that were not previously induced by phenobarbital (PHE) exposed for 12 h, and increased it in PHE-induced cells exposed to the highest CYN concentration (10 µg/L). After 24 h of exposure, however, LPO experienced an increase in both cell types only at 10 µg/L CYN.

In the cellular environment, ROS increases are counteracted by enzymatic and non-enzymatic defensive mechanisms. In the first group, the enzymes superoxide dismutase (SOD), catalase (CAT), and glutathione peroxidase (GPx), among others, play an important role. SOD, CAT, GPx, Glutathione S-transferase (GST), and glucose-6-phosphate dehydrogenase (G6PDH) were not altered in *Hoplias malabaricus* hepatocytes that were exposed to up to 100 μg/L CYN for 72 h [63]. Different results were obtained by Poniedziałek et al. (2015) in human lymphocytes. SOD and CAT levels decreased and GPx activity increased. These effects were mainly observed after 6 h of exposure. Liebel et al. [41] also investigated GST activity in a HepG2 cell line and found an increase. Moreover, a higher level of Nrf2 (a transcription factor that regulates the expression of antioxidant enzymes) in toxin-treated rat primary hepatocytes after 48 h was observed by López-Alonso et al. [38].

From a general perspective, the variability of the results that were obtained for a particular biomarker (GSH, ROS, etc.) is high. These differences could be due to the different experimental models (primary cells or cell lines of various origins), CYN concentrations, or exposure periods that were employed. Moreover, given that CYN is also an environmental contaminant, it is noteworthy that few reports are available with fish *in vitro* models [34,35,61,63].

Another point to highlight is that all of these studies have been performed with a CYN pure standard or CYN isolated from *Cylindrospermopsis raciborskii*. The differences between the toxic effects that were induced by pure and extracted CYN have not been systematically studied so far. It is known, however, that in the case of a different cyanobacterial toxin, microcystins, extracts may contain other compounds that can influence the toxicity observed [65]. Therefore, it would be necessary also to test the effect of an extract from a non-CYN-producing culture in order to establish the contribution of these substances to the final response. Also, other oxidative stress biomarkers (protein or DNA oxidation) have not yet been studied, nor has the effect of different chemoprotectants on oxidative stress biomarkers [66]. In this regard, only López-Alonso et al. [38] observed that resveratrol partially reduced the cytotoxicity that was induced by CYN in primary rat hepatocytes. These authors argued that oxidative stress is involved in the cytotoxicity induced by CYN at lower concentrations in primary rat hepatocytes. The explanation was that the low toxin concentrations and long exposure times induced apoptosis, while in the case of necrosis induction the insult to the cells produced by the toxin could be of such a magnitude, and cell death so rapid, that oxidative stress could not be observed.

From all of these results (and also from those obtained *in vivo* and not considered here), it has been shown that CYN induces oxidative stress. It should be of interest to investigate the repercussion that this can have on human and environmental health, as ROS generation may account for the increased risk of cancer development in the aged [67].

### 4.3. Genotoxicity

Besides being considered as a cytotoxic toxin, CYN has also been described as genotoxic [68]. Several studies imply that it is pro-genotoxic [69], although the genotoxicity of CYN (and/or its metabolites) is still controversial [7]. Genotoxic and even carcinogenic effects of CYN have been reported *in vivo* in mice by several authors [70,71], the liver being the most affected organ. This suspicion is based on the nucleotide structure of CYN, which contains potentially reactive guanidine and sulfate groups [70,72]. The presence of uracil led researchers to suggest a possible interaction with nucleic acids [27].

With regard to *in vitro* studies, various assays have been performed (Table 5) [26,27,28,35,53,60,63,72,73,74,75,76,77,78,79,80,81,82], mainly in mammalian systems, which demonstrated the pro-genotoxic activity of CYN.

It is important to note that no single genotoxicity test is capable of detecting all relevant genotoxic agents, and therefore various international organizations recommend a test battery of genotoxicity assays to elucidate the genotoxic potential of a biotoxin (such as CYN), xenobiotics, new medical devices, or substances in contact with food, etc. The data compiled in Table 5 indicate that, in general, there are few studies, and only one work that has been performed regarding the mutagenic profile of CYN in bacteria [76], showing negative results. In mammal cells, the alkaline comet assay is the genotoxic assay that is most frequently used to test a pure standard of CYN [74,75,78,82], or CYN isolated and purified from crude extracts obtained from cyanobacterial cultures (usually from *C. raciborskii* [26,73,80]. After applying the comet assay in various metabolic cells, most of the results showed a positive response of CYN, increasing the comet tail length, area, or moment, while cell alterations, but no DNA fragmentations were induced by CYN in metabolism-deficient Chinese hamster ovary K1 (CHO-K1) cells [73]. At molecular level, research has been carried out on changes in the expression of genes that are involved in the response to DNA damage induced by CYN alone on HepG2 cells [60,74,79], and, more recently, by a binary mixture of cyanotoxins CYN and MC-LR [82], indicating the mechanisms involved (oxidative stress, etc.). Further studies in this direction are needed in different experimental models.

Simultaneously, with the comet assay, the *in vitro* cytokinesis-block micronucleus assay (CBMN) is being increasingly used in the evaluation of CYN [53,72,74,75,82], rather than the *in vitro* chromosome aberration (CA) assay, which applied only in CHO-K1 cells (Lankoff et al., 2007) [28]. This may be due to some advantages that are offered by the MN test, such as the high number of analyzable cells, simplicity of the technique, possible automation, and the ability to detect aneugens more accurately [83,84].

It is important to note that, to the best of our knowledge, the enzyme-modified comet assay, using endonuclease III (Endo III) and formamidopyrimidine DNA glycosylase (Fpg) to detect oxidation of pyrimidines or purine DNA bases, respectively [85], has rarely been employed to evaluate the role of this mechanism in the genotoxic potential of CYN. Only one study has investigated the oxidation of purine bases (Fpg assay) by CYN [61]. In addition, the mouse lymphoma gene mutation assay (MLA) (OECD 476) [86], which is preferred because it detects the broadest set of genotoxic mechanisms—such as chromosomal, gene, base pair substitutions, and frame-shift mutations [87,88]—that are associated with carcinogenesis activity, has not been performed either, despite the indications of CYN carcinogenicity for humans) [7,69].

In comparison to mammals, the genotoxicity of CYN in fish has been poorly studied, and contradictory results have been found by the alkaline comet assay in various cells from several fish species [35,63,77]. A positive response has been shown by CYN in the CBMN assay on a leucocyte cell line from *Cyprinus carpio* L. [77].

Recently, some studies have evaluated the potential mutagenicity/genotoxicity of CYN in combination with other cyanobacterial toxins, mainly MC-LR [76,82], because in real life, organisms are exposed to mixtures of several biotoxins, rather than to a single compound [89], and following the recommendations that are given by international organizations, such as the European Food Safety Authority (EFSA) [19].

More detailed information about the results and conclusions stated in the genotoxic studies compiled in this review is provided below.

The only evidence from bacterial test systems (Ames test) indicated that CYN pure standard was not mutagenic toward the bacterial strains (*S. typhimurium* and *E. coli*) assayed up to a concentration of 10 µg/mL, a concentration higher than those considered ecotoxicologically relevant [76]. Negative responses were also found for the pure standard solutions of the cyanotoxins MC-LR and anatoxin-a under the conditions assayed. Neither an increase in the number of revertants nor an inhibition of the growth of bacteria was observed, with or without metabolic activation. Similarly, there were negative results after exposure of bacteria to the mixture of pure toxins. By contrast, extracts that were obtained from cyanobacterial bloom-forming cells harvested from environmental waters were evidently mutagenic, mainly against *S. typhimurium* TA100 strain, and only contained CYN in a low concentration (0.89 µg/L). It was concluded that neither CYN nor other cyanotoxins that were tested were responsible per se for the observed mutagenicity of the extracts, and perhaps some other components of cyanobacterial extracts were responsible for the induction of mutations. The authors suggested that, while it can be stated that CYN and MC-LR are not mutagenic for the bacterial strains that are used, there are many reasons for considering these compounds as mutagens for eukaryotic cells. In addition, the metabolic activation enzyme system (S9 fraction) derived from rat livers employed in the Ames test may differ from the metabolism occurring in human cells [76]. Further studies are needed to confirm these preliminary results, especially in the case of CYN, and to elucidate potential synergistic interactions between cyanotoxins.

In mammalian systems, Humpage et al. [72] showed that CYN could induce micronuclei (MN) *in vitro* in human lymphoblastoid WIL2-NS cells, and this effect was mainly linked to an aneugenic effect, and, to a lesser extent, to a clastogenic one. These authors suggested that CYN acts to induce cytogenetic damage using two mechanisms: one at the level of the DNA to induce strand breaks, producing acentric fragments and giving rise to centromere-negative micronuclei; the other at the level of the kinetochore/spindle function, to induce loss of whole chromosomes owing to malsegregation of chromosomes during anaphase, which may possibly be explained by the known effects of CYN on protein synthesis. Metabolism of CYN by WIL2-NS cells is yet to be confirmed, so the involvement of CYN metabolites is not clear [69].

By contrast, when the comet assay was employed in CHO-K1 cells that were exposed to CYN isolated and purified from cultures of *C. raciborskii*, no DNA damage was detected 24 h after treatment, although inhibition of cell growth was reported, and also blebbing and rounding of the cells, linked to cytoskeletal reorganization but not to apoptosis [73]. The authors concluded that CYN did not react directly with DNA, but they pointed to the potential role of its metabolization in the generation of genotoxic products. As no exogenous metabolic activation was used in this study, the lack of DNA damage could be due to the low metabolizing enzyme activity of these cells. This was the first time that the need for further research taking into account the importance of CYN was highlighted.

In this context, to understand the role of CYP450-activated CYN metabolites in the *in vitro* genotoxicity of CYN, Humpage et al. [26] applied the comet assay in primary mouse hepatocytes, both in the presence and in the absence of CYP450 inhibitors, such as omeprazole and SKF525A. The direct assay revealed a statistically significant concentration-dependent increase in comet tail length, area, and moment in cells that were treated for 18 h with CYN (0.05–0.5 µM), and significant DNA fragmentation at a concentration as low as 0.05 µM. The genotoxicity of CYN at subcytotoxic concentrations, below the EC30 where cell death-related DNA digestion should not be detectable [90], suggests that it is a specific and primary effect of CYN. The fact that CYP450 inhibitors, such as omeprazole (100 µM, an inhibitor of CYP 3A4/2C19) and SKF525A (50 µM, a broad-spectrum CYP inhibitor), completely inhibited the genotoxicity that was induced by CYN indicated that CYP450-derived metabolites of the toxin are responsible for its genotoxicity [26].

Other experiments performed to know whether the metabolism could be a prerequisite for CYN-genotoxicity were carried out in CHO-K1 cells, and no chromosome aberrations (CA), with or without metabolic activation (S9 fraction), were detected [28]. The results revealed that CYN was not clastogenic in CHO-K1 cells, irrespective of S9 fraction-induced metabolic activation. However, the toxin significantly decreased the frequencies of mitotic indices and cell proliferation, irrespective of the metabolic activation system. This lack of genotoxicity of CYN confirmed the previous results that were found in the comet assay in the same cell line [73], and showed that, despite the use of metabolic activation (S9 mix, post-mitochondrial supernatant, known to be a potent enzymatic inducer), the frequency of CA was not affected by CYN. Consequently, CYN itself and S9-derived metabolites of the toxin are non-clastogenic under these experimental conditions in CHO-K1 cells [28]. Various factors may be responsible for the discrepancy between the cytogenetic assay results and the comet assay study performed with hepatocytes [26]: (1) the lack of an appropriate metabolic system, because the liver S9 elevates the levels of several CYN metabolizing enzymes, but it does not cover their total spectrum (e.g., CYP1A1 or CYP2E1 are low or inactivated in S9 fraction); (2) diffusion pathways are longer for externally generated active metabolites; (3) some genotoxic metabolites may be formed only within specific target cells; (4) the doses of CYN that were efficient to induce DNA single-strand breaks visible in the comet assay were too low to induce CA; (5) the cytotoxic property of CYN may be a confounding factor in the comet assay, giving false positive results; and, (6) differences in CYN uptake in different cell lines. In conclusion, the metabolic activation of CYN influenced the cytotoxicity of CYN by increasing the susceptibility to necrotic cell death, and the positive comet assay results observed by others could be due to cytotoxicity rather than to genotoxicity. Although CYN did not induce DNA damage and CA in CHO-K1 cells, it affected the microtubular structure in this cell line, which could disrupt spindle or centromere function and may lead to loss of whole chromosomes [33,69].

In contrast, CYN induced MN formation in two human cell lines, hepatocyte (HepaRG) and enterocyte (Caco-2) cell lines, models of CYN target organs [53]. After exposure to 1.25–1.5 µg CYN/mL, significant increases in MN (3-fold above controls) in both differentiated and undifferentiated Caco-2 cells were detected. No increase in MN formation was detected in undifferentiated HepaRG cells, and a positive response at 0.06 µg CYN/mL in differentiated HepaRG cells (1.8-fold) was reported. This last genotoxic effect was found in a similar dose range in primary hepatocytes by the comet assay, suggesting that the CYN-metabolizing capabilities of differentiated HepaRG may be similar to hepatocytes. There were differences in genotoxicity in the two differentiated cells, the increase in MN frequency being greater in Caco-2 cells. The assay was also performed using the inhibitor of CYP450, ketoconazole, which is widely known to inhibit CYP3A4, a potent inhibitor of CYP1A1, and a moderate inhibitor of CYP2C, CYP1A2, and CYP2D6. Ketoconazole strongly protected undifferentiated Caco-2 cells and reduced cytotoxicity and induction of MN to 50%, in agreement with the findings reported by Humpage et al. [26] with omeprazole. However, the pretreatment with ketoconazole showed no effect on MN-induction by CYN in differentiated HepaRG cells. Therefore, it seems that CYN genotoxicity is mediated through its metabolites, suggesting that this toxin is a progenotoxin, and that minor CYP isoforms may play a role in its metabolic activation [69]. Until now, there was only indirect evidence for the formation of reactive CYN metabolites; consequently, the reduction of CYN toxicity in the presence of CYP inhibitors could be due to alternative pathways [48].

On the other hand, toxicogenomic approaches could elucidate CYN toxicity mechanisms [74,75,79,82]. In human hepatoma HepG2 cells, at non-cytotoxic concentrations, Štraser et al. [74] indicated that CYN induced DNA breakage, and a dose-dependent increase in the frequencies of MN, nuclear buds (NBUD), and nucleoplasmic bridge (NPB) formation, which were associated with upregulation of DNA damage responsive genes CDKN1A, GADD45α, and MDM2. The authors also showed that CYN induced upregulation of some genes presumably involved in CYN metabolism, such as CYP1A1 and CYP1A2. These results are in agreement with previous studies in primary hepatocytes [26], indicating a similar metabolizing capacity of both *in vitro* models; and, with the induction of MN by CYN in the three human cell lines mentioned above, the lymphoblastoid cell line WIL2-NS [72], liver HepaRG cells, and colon-derived Caco-2 cells [53]. It is noteworthy that Štraser et al. [74] provided the first evidence that exposure to CYN induced transcription of CYP1A1 and 1A2 isoforms, supporting the assumption that they are involved in CYN metabolic activation to genotoxic intermediates. Moreover, as CYN induced NBUD and NBP formation, which correlated with increased MN formation, these authors indicated that CYN induced complex genomic alterations, including gene amplification and structural chromosomal rearrangements. The toxicogenomic analysis indicated the upregulation of DNA damage responsive genes, confirming the previous study by Bain et al. [27], who detected upregulation of CDKN1A, GADD45α, MDM2, and BAX in HepG2 cells and in human dermal fibroblasts that are exposed to CYN.

In the same cell line, it was also demonstrated for the first time that CYN caused double-strand breaks (DSBs) and had impacts on the cell cycle, providing evidence that the toxin is a directly acting genotoxin [60]. Similarly, in human peripheral blood lymphocytes (HPBLs), Žegura et al. [75] found, after exposure to pure standard CYN, that the toxin induced the formation of DNA single-strand breaks (comet assay) and a time- and dose-dependent increase in the frequency of MN and NBUD, and only a slight increase in NPB, confirming the previous results that were reported for HepG2 cells. The effects of CYN on mRNA expression of selected genes was again similar to the effects found in HepG2 cells: the genes involved in CYN metabolism (CYP1A1 and CYP1A2) were upregulated, indicating that they are involved in CYN metabolic activation, although other CYP isoforms might also be implicated. In addition, CYN induced significant upregulation of the P53 gene, as well as its downstream regulated genes (MDM2 and GADD45α), apoptosis genes (BCL-2 and BAX), and, for the first time, some stress responsive genes (GPX1, SOD1, GSR, GCLC). Subsequently, these authors confirmed the time-dependent upregulation of the growth arrest and DNA-damage inducible genes (GADD45α and GADD45β), and the genes involved in DNA damage repair (XPC, ERCC4, and others), indicating cell-cycle arrest and induction of nucleotide excision and double-strand break repair [79]. In relation to detoxification response, evidence for the involvement of phase I and phase II enzymes was also demonstrated. After longer exposure (24 h), CYN could induce the possible depletion of glutathione and minor oxidative stress, as indicated by the upregulation of some genes—catalase gene (CAT), thioredoxin reductase (TXNRD1), and glutamate-cysteine ligase (GCLC)—although other genes were not induced. This minor role of oxidative stress in the genotoxicity of non-cytotoxic concentrations of CYN was also confirmed in the same HepG2 cells, because non-oxidative DNA damage was detected after the application of the enzyme-modified comet assay (Fpg digestion) [60].

In relation to fish, despite the common exposure of fish in natural environments and fish farms to cyanotoxins, there are only four studies on the genotoxic effects of CYN, yielding contradictory results, as mentioned earlier [35,61,63,77]. Cell-type and interspecific CYN toxicity differences may occur, because, in comparison to the concentration-dependent DNA damage reported in mammal cells [28,72], DNA breaks were not found in fish hepatocytes that were exposed to the same concentrations of CYN [35]. In this work, hepatocytes of *P. lineatus* that were exposed to environmentally relevant concentrations of CYN (0.1–10 μg/L) significantly decreased cell viability, there were changes in some oxidative stress biomarkers, but no significant alterations in DNA strand breaks were found by the comet assay. Similar negative results on DNA damage were reported in hepatocytes of *H. malabaricus* [63]. By contrast, an increased amount of DNA strand breaks was observed in common carp (*C. carpio*) blood leucocytes exposed to pure CYN (0.5 μg CYN/mL), not connected with cell death, although to a lesser extent, in comparison to the cyanotoxin MC-LR, which was the most toxic cyanotoxin [77]. On the other hand, in the fish CLC cell line (carp leukocyte culture cell line) CYN exposure (0.1–1.0 μg CYN/mL) induced MN, and for the first time, oxidative DNA damage was found by detection of the oxidation product 8-oxo-7,8-dihydro-2′-deoxyguanosine (8-OHdG) [61]. The effects exerted by CYN on CLC might be associated with oxidative stress and may result in genotoxic effects. While the increased level of 8-OHdG can be explained by the ROS production that was observed, the mechanism of MN induction was partially due to the clastogenic activity of the toxin. Although the vast majority of MN detected in binucleated CLC cells was much smaller than a quarter of the size of normal nuclei, the authors cannot speculate that they contained only DNA fragments, because the chromosome size in carp is heterogeneous. They concluded that CYN acts both as a clastogen and as an aneugen, as in mammal cell lines.

Recently, the genotoxic potential of binary mixtures of CYN and MC-LR has been studied for the first time, owing to the ubiquitous and simultaneous presence of both genotoxic cyanotoxins in the aquatic environment [82]. In this study, HepG2 cells were exposed to different doses of CYN (0.01–0.5 μg/mL), a single dose of MC-LR (1 μg/mL), or to several combinations of them. After 24 h exposure, CYN individually induced DNA strand breaks (comet assay) and genomic instability as measured by the CBMN assay. The MC-LR/CYN mixture induced both genotoxic injuries, but in the case of strand breaks to a lesser extent than CYN alone. The findings obtained by the comet assay confirmed previously published data that showed that MC-LR induced DNA strand breaks after short-term exposure, probably owing to oxidative stress (oxidation DNA bases) [69,91], while CYN induces DNA damage after longer exposure in metabolically active cells [26,74]. Lower DNA damage was detected with the mixture, and this antagonistic effect could be explained by the attenuated DNA repair that is produced by MC-LR [92,93]. The induction of genomic instability by CYN corroborated that this toxin induces MN formation, previously reported in metabolically active [53,74], while MC-LR alone did not induce MN formation at a low concentration [93]. The fact that CYN/MC-LR mixtures induced similar genomic instability in comparison to CYN alone indicates that MC-LR has no effect on CYN. Moreover, mRNA expression of selected genes after 4 and 24 h of exposure to individual cyanotoxins, and their combinations was performed by qPCR for the first time. The changes in the expression of some genes involved in xenobiotic metabolism, belonging to the group of phase I metabolism (CYP1A1, CYP1A2, CYP1B1, CYP3A4) and phase II (GSTA1, NAT2, UGT1A1), genes that are involved in immediate-early response/signaling (FOS, JUNB, MYC, and TGFB2), and the transcription of genes involved in DNA damage response (CDKN1A, CHEK1, ERCC4, GADD45A, MDM2, and TP53) observed with the CYN/MC-LR mixture were not different from those induced by CYN alone. All of these results indicate that CYN has higher genotoxic effects than MC-LR in the MC-LR/CYN mixture. MC-LR has no effect on CYN-induced deregulation of the selected genes reflecting the mechanisms of its pro-genotoxic activity.

Overall, more studies in different human cell lines are needed to confirm these findings after exposure to a mixture of CYN with other cyanotoxins. Moreover, the application of complementary mutagenic/genotoxic assays would be very useful, assays, such as: (1) the Ames test in bacterial systems to confirm the only study published; (2) the enzyme-modified comet assay to know whether DNA oxidation is involved in the induction of strand breaks; and, (3) the MLA assay, to elucidate whether CYN alone, or the combination CYN/MC-LR, is able to induce mutations in mammalian cells (L5178Y/Tk ± cells). Furthermore, toxicogenomic and proteomic studies would help to elucidate the mechanisms of CYN genotoxicity.

### 4.4. Immunotoxicity

The effect of CYN on the immune response is not well studied, although it can potentially affect cells of the immune system and alter its function, as has been reported *in vivo* in rodent models [55,94]. *In vitro*, the studies are very scarce, and the first work of CYN effects was reported using human peripheral blood lymphocytes from different but healthy donors [95]. At the highest concentration of CYN assayed (1 µg/mL), a significant inhibition of lymphocyte proliferation after 24 h of exposure was reported, and it resulted in inhibition of thymidine incorporation. Following these investigations, in human lymphocytes that were exposed to purified CYN isolated from a *C. raciborskii* culture (0.01–1.0 µg/mL), the authors demonstrated its antiproliferative activity during different phases of their activation. The highest concentration induced the most significant inhibition (over 90% when compared to unaffected cells) at the beginning of their activation. Moreover, a cell-cycle arrest at G0/G1 and prolonged S phase in lymphocytes undergoing activation and significant apoptosis inducement in activated cells were also detected [40]. It was suggested that DNA damage may be a primary mechanism of CYN action in lymphocytes, which is supported by DNA single-strand breaks, as observed by Žegura et al. [75]. These findings indicated that CYN could be classified as a potential immunotoxicant, and that potentially it could reduce abilities to fight pathogenic microorganisms or malignant cells [40].

Subsequently, these authors investigated whether these effects were mediated by alteration in the ROS level and oxidative stress of human-derived lymphocytes [62]. At the same concentrations of CYN mentioned above (0.01–1.0 µg/mL), the toxin induced a concentration- and time-dependent increase of H_2_O_2_ content, and also changes in several oxidative biomarkers, such as decreased activities of SOD and CAT, elevated level of GPx, and induction of LPO. All of these findings help to elucidate that the oxidative stress that is triggered by CYN in human cells is involved in the reported cyanotoxin-induced DNA damage, cell-cycle arrest, and apoptosis previously reported.

Neutrophils, an important part of the immune system, are highly specialized white blood cells that protect against infection in a non-specific manner. CYN (0.01–1.0 µg/mL) can affect the function of human peripheral blood neutrophils during 1 h exposure. CYN had no significant effect on the phagocytic activity of neutrophils, and no apoptotic or necrotic action was revealed [96]. However, it was found that CYN significantly altered neutrophil oxidative burst, a key process in pathogen elimination.

In addition to the immunomodulatory action of CYN on T lymphocytes and neutrophils, the potencies of metabolites that are produced by non-CYN-producing strains of *C. raciborskii* have been investigated in both human cells, and the observed effects were very similar to those that are induced by CYN [97]. After short-term treatments, the extracts altered viability of cells by increasing necrosis and apoptosis in neutrophils, and elevated apoptosis in lymphocytes, whereas no effects were observed with CYN. In general, lymphocytes appeared to be more resistant than neutrophils. T lymphocytes that were exposed for 72 h to *C. raciborskii* extracts resulted in a decrease of proliferation, and exposure to CYN (1.0 µg/mL) caused lymphocyte proliferation that later decreased. The effect of the extracts on T lymphocyte proliferation was not as pronounced as for CYN, suggesting that the cells can partially overcome the injuries that are induced by *C. raciborskii* exudates, or the metabolites could be degraded owing to their lower stability in comparison to CYN. This *in vitro* study indicated for the first time that extracts of *C. raciborskii* contained compound(s) (not identified yet) capable of altering the function of the human immune system.

## 5. Concluding Remarks

The higher number of reports on *in vitro* CYN toxicity studies deals with basal cytotoxicity aspects. This finding is not surprising taking into account that *in vitro* methods are widely used for screening purposes. The second aspect most frequently evaluated is genotoxicity. This is in accordance with the great importance that genotoxicity testing has nowadays. In view of the adverse consequences of genetic damage to human health, the assessment of mutagenic/genotoxic potential is a basic component of chemical risk assessment. Currently, genotoxicity testing is included in the first step of tiered toxicity evaluation approaches for various kinds of compounds, such as additives [98] or food contact materials [99].

In order to obtain a better understanding of the toxicity that is exerted by CYN, the toxicokinetics of CYN should be studied further. In this context, the mechanism of cellular uptake of CYN should be completely elucidated. This would also make it possible to discern the target organ and propose potential therapeutic agents for CYN intoxication. Moreover, the metabolites of CYN have not been described so far. For the main toxicity mechanisms that are considered in this review, potential data gaps have already been identified (see Section 4.1, Section 4.2, Section 4.3 and Section 4.4). From a general point of view, various remarks can be made. For example, no studies on the effects of extracts from non-CYN-producing cyanobacterial strains have been identified, although it has been demonstrated that extracts from non-MC-producing strains also show toxic effects. There is no toxicological information about analogs other than CYN. Also, the near absence of studies dealing with cyanobacterial mixtures needs to be highlighted, and these investigations should be prioritized, as already indicated by EFSA [19]. Moreover, there are other new emerging toxicity effects that are attributable to CYN, such as neurotoxicity or immunotoxicity, which have scarcely been investigated by *in vitro* methods. Therefore, *in vitro* toxicity testing can still be very useful to complete knowledge about the toxic profile of CYN and its related compounds.

## Figures and Tables

**Table 1:** *In vitro* cytotoxicity studies performed with Cylindrospermopsin (CYN).

Toxin/Cyanobacteria	Experimental Model	Assays Performed	Exposure Conditions Concentration Ranges	Main Results	Reference
Purified extract from *Cylindrospermopsis raciborskii*	Primary rats hepatocytes	Lactate dehydrogenase (LDH) activity	0.5–5 µM for 0–18 h	After 18 h of incubation with 3.3 and 5 µM, significant cell death (40% and 67%, respectively) was found. No measurable cell lysis within the first 12 h of exposure to CYN, although slight signs of rounding were observed.	[22]
Commercial CYN pure standard	Primary rat hepatocytes and KB cells	3-(4,5-dimethylthiazol-2-yl)-2,5 diphenyltetrazolium bromide (MTT) assay	0–10,000 ng/mL for 0, 24, 48, 72 h	Toxic effects were observed at after 72 h, being the LC50 40 ng/mL in the case of exposure to rat hepatocytes while in KB cells it was 200 ng/mL.	[23]
Purified extract from *Cylindrospermopsis raciborskii*. Synthetic CYN and analogues: racemic CYN (RAC-CYN), CYN-DIOL, AB-MODEL, Epi-cylindrospermopsin (EPI-CYN), and EPI-DIOL	Primary rats hepatocytes	LDH release	CYN: 0.16–10 µM RAC-CYN: 1.25–20 µM CYN-DIOL: 0.16–5 µM EPI-CYN: 0.075–12.5 µM EPI-DIOL: 0.1–50 µM 19 h	When hepatocytes were exposed to 20 µM of RAC-CYN cell death increased from 14% to 23%, while in the case of CYN-DIOL cell death enhanced up to 38%. Similar results were observed in exposures to 10 µM CYN, 12.5 µM EPI-CYN and 50 µM EPI-DIOL with increases of 33.4%, 38.5% and 35%, respectively.	[24]
Purified extract from *Cylindrospermopsis raciborskii*	Primary rat hepatocytes; Caco-2 and HepG2 cells	MTT assay	24 h to primary rat hepatocytes 48 h to permanent cells lines	None of the 7 isolates of *C. raciborskii* contained CYN; however, they were all toxic. The methanolic extracts were generally more toxic than the aqueous extracts.	[25]
Commercial CYN pure standard	Primary mouse hepatocytes	LDH release	0.05–25 µM for 18, 21, 24 h	Time- and concentration- dependent increases in LDH leakage was observed after exposure to CYN. The EC50 at 18 h was 0.47 μM. The concentration response was very steep, with concentrations of 1 μM and above producing greater than 75% LDH leakage within 18 h whereas concentrations below 0.1 μM had no effect.	[26]
Commercial CYN pure standard	HDF, HepG2, and Caco-2 cells	MTS assay and LDH leakage	0.1–5 μg/mL CYN for 24, 48, 72 h	Although it was not possible to calculate the IC50 for the MTS assay due to lack of data for higher concentrations, a time-dependent effect was observed in all three cell types. However, no effect was observed in the LDH assay in rHepG2 and Caco-2 cells, but HDF cells reached 30% of the lysed controls at concentrations above 1 μg/mL CYN (2.4 μM) after 72 h.	[27]
Purified extract from *Cylindrospermopsis raciborskii*	CHO-K1 cells	Annexin V- fluorescein isothiocyanate/propidium ionide (FITC) apoptosis detection kit	0.05–2 µg/mL for 3, 16, 21 h	CYN increases the frequency of necrotic cells in a dose and time-dependent manner, but very slight impact on apoptosis was observed. In addition, when cells are metabolic activated the susceptibility to necrotic cell death increases, whereas it has no impact on apoptosis.	[28]
Purified extract containing CYN and deoxyCYN	HepG2, BE-2, Caco-2, MNA, HDF	Trypan blue exclusion test (TBET) and MTS assays	0.1–5 µg/mL for 24, 48, 72 h	Both CYN and deoxyCYN exerted toxic effects to all exposed cells in a concentration and dependent way, being deoxyCYN slightly less cytotoxic than CYN.	[29]
Commercial CYN pure standard	Primary human granulosa cells	MTT assay	0–1 µg/mL for 2, 4, 6, 24, 48, 72 h	No effect was recorded in cells exposed up to 1 µg/mL in short 2–6 h exposures. However, cell viability decreased in a concentration-dependent way at longer exposures (24–72 h).	[30]
Commercial CYN pure standard	C3A, HepG2, NCI-87, HCT-8, HuTu-80, Caco-2, and Vero cells	MTT assay and LDH leakage	0.4–66 µM for 1, 2, 4, 6, 24 h	The 24 h IC50 for CYN cytotoxicity was set at 1.5 µM for hepatic cell lines (C3A and HepG2 cells), while for colonic cells (Caco-2) the IC50 was 6.5 µM. Similar onset was found in hepatic cells (C3A) in long-term exposures up to 7 days. No recovery of the toxicity caused by CYN was evidenced in C3A cells after exposure for 1–6 h.	[31]
Commercial CYN pure standard	Vero-GFP cells	MTS (3-(4,5-dimethylthiazol-2-yl)-5-(3-carboxymethoxyphenyl)-2-(4-sulfophenyl)-2H-tetrazolium) assay	0.1–100 µM for 4, 24 h	The IC50 found for CYN after 24 h was 5.9 µM. The use of other protein inhibitors indicated that the toxicity exerted by CYN was not only related to protein synthesis mechanism but other effects may contribute to the toxicity observed.	[32]
Commercial CYN pure standard	CHO cells	Annexin V-FITC assay	0.1–10 µM for 12, 18, 24, 48 h	CYN cause apoptosis at low concentrations (1–2 µM) and over short exposure periods (12 h). Necrosis was observed at higher concentrations (5–10 µM) and following longer exposure periods (24 or 48 h).	[33]
Commercial CYN pure standard	PLHC-1	Protein content (PC), neutral red uptake (NRU) and MTS assay	0.3–40 µg/mL for 24, 48 h	Cytotoxic effects were observed in all the endpoints assayed in a time and concentration-dependent manner. Regarding the EC50 values, the most sensitive endpoint was PC for 24 h of exposure, with an EC50 of 8 µg/mL, and MTS assay for 48 h with an EC50 of 2.2 µg/mL.	[34]
Purified extract containing CYN	Primary *Prochilodus lineatus* hepatocytes	NRU	0.1–10 µg/L for 72 h	Cell viability decreased 8% in hepatocytes exposed to 0.1 and 1 µg/L. However, at the highest concentration assayed (10 µg/L) no significant change was observed in comparison to the control.	[35]
Commercial CYN pure standard	Caco-2	PC, NRU and MTS assays	0.3–40 µg/mL for 24, 48 h	The most significant endpoint was MTS assay. This endpoint revealed significant cytotoxicity in Caco-2 cells exposed to all concentrations assayed except for the lowest concentration after 24 h. The EC50 were 2.5 µg/mL for 24 h and 0.6 µg/mL for 48 h.	[36]
Commercial CYN pure standard	HUVEC	PC, NRU and MTS assays	0.3–40 µg/mL for 24, 48 h	The higher cytotoxic effects were observed in NRU. Very low rates of cell viability were reported at 40 µg/mL, being 20% and 3% after 24 and 48 h, respectively. Similarly, low EC50 were found, 1.5 µg/mL for 24 h and 0.8 for 48 h.	[37]
Commercial CYN pure standard	Primary rat hepatocytes	Alarm blue assay	10–360 nM for 24, 48 h	CYN reduced cell viability in hepatocytes exposed to 90, 180 and 360 nM CYN. The two higher concentrations (180 and 360 nM) decreased cell viability around 50% after 48 and 24 h, respectively.	[38]
Commercial CYN pure standard	Caco-2 and Clone 9 cells	Alarm blue assay	0.1–10 µM for 8, 10, 12, 24, 48, 72 h	No cytotoxicity was observed for Caco-2 cells exposed to CYN up to 72 h. However, a time and concentration-dependent decrease in viability of Clone 9 cells exposed to CYN in comparison to the controls.	[39]
Commercial CYN pure standard	Primary human T-lymphocytes	FAM caspase activity kit	1 µg/mL for 6, 24, 48 h	The viability of human T-lymphocytes decreased in a concentration and time dependent way. Significant decreases were observed in exposure to 1 µg/mL, with the highest alterations observed after 24 h of exposure.	[40]
Purified extract containing CYN	HepG2	NRU and MTT assay	0.001–100 µg/L for 4, 12, 24, 48 h	CYN was not toxic to HepG2 cells after 48 h of exposure, except for the higher concentration (100 µg/L) with a decrease of 11%. At concentrations bellow 10 µg/L cell viability increased.	[41]
Synthetic CYN analogues (11a, 11b, 11c and 22), 1 and guanidinoacetate (GAA)	human neutrophils	MTT assay	2.0 μg/mL for 1 h	The general toxicity decreased in the following order: 11c > 11a > 1 > 11b > 22 > GAA. No remarkable toxic effect was observed for the two last compounds (22 and GAA).	[42]

Abbreviations: BE-2 (Caucasian bone-marrow neuroblastoma cell line); Caco-2 (human colorectal adenocarcinoma cell line); CHO (Chinese hamster ovary cell line); CHO (a subclone from the parental CHO cell line); cylindrospermopsin (CYN); C3A (human hepatocellular carcinoma); effective mean concentration (EC50); guanidinoacetate (GAA); HCT-8 (human ileal adenocarcinoma); HDF (human dermal fibroblast cell line); HepG2 (human liver hepatocellular carcinoma cell line); HuTu-80 (human duodenal adenocarcinoma); HUVEC (human vascular endothelium cell line); IC50 (inhibitory mean concentration); KB (human cervix carcinoma); MNA (mouse neuroblastoma cell line); MTS (3-(4,5-dimethylthiazol-2-yl)-5-(3-carboxymethoxyphenyl)-2-(4-sulfophenyl)-2H-tetrazolium); MTT (3-(4,5-dimethylthiazol-2-yl)-2,5 diphenyltetrazolium bromide); NCI-N87 (human gastric carcinoma); PLHC-1 (*Poeciliopsis lucida* hepatocellular carcinoma cell line); SHE (Syrian hamster embryo cell line); Vero (African green monkey kidney).

**Table 2:** *In vitro* morphological studies dealing with CYN.

Toxin/Cyanobacteria	Experimental Model	Microscopy Used	Exposure Conditions Concentration Ranges	Main Results	Reference
Purified extract containing CYN and deoxyCYN	HepG2, BE-2 and MNA cells	Light microscope	0.5–5 µg/mL for 24, 48 h	The BE-2 and MNA cells underwent shrinkage and cell rounding at 2.5 and 5 µg/mL, respectively. These findings indicated apoptosis process.	[29]
Commercial CYN pure standard	SHE cells	Light microscope	1 × 10^−7^–1 × 10^−3^ ng/mL for 7 days	CYN induced morphological cell transformation after 7 days of treatment with CYN (1 × 10^−7^–1 × 10^−2^ ng/mL), with nuclear enlargement. The morphologically transformed phenotype also showed loss of contact inhibition and density-dependent inhibition of cells.	[42]
Commercial CYN pure standard	Caco-2 cells	Light and electron microscopes	0.625, 2.5 µg/mL for 24, 48 h	The most remarkable ultrastructural changes were lipid degeneration, mitochondrial damage and nucleolar segregation with altered nuclei.	[36]
Commercial CYN pure standard	HUVEC cells	Light and electron microscopes	0.3–40 µg/mL for 24, 48 h	The main findings observed were nucleolar segregation with altered nuclei, degenerated Golgi apparatus, increases in the presence of granules and apoptosis.	[37]
Commercial CYN pure standard	Clone 9 cells	Light microscope	5 µM for 24, 48 h	After 24 h of treatment with CYN no discernible effect was observed, although after 48 h signs of damage and detachment of cells were reported.	[39]

Abbreviations: BE-2 (Caucasian bone-marrow neuroblastoma cell line); Caco-2 (human colorectal adenocarcinoma cell line); Clone 9 (*Rattus norvegicus* epithelial liver cell line); cylindrospermopsin (CYN); deoxycylindrospermopsin (deoxyCYN); HepG2 (human liver hepatocellular carcinoma cell line); HUVEC (human vascular endothelium cell line); MNA (mouse neuroblastoma cell line); SHE (Syrian hamster embryo cell line).

**Table 3:** *In vitro* toxicokinetics studies performed with CYN.

Toxin/Cyanobacteria	Experimental Model	Assays Performed	Exposure Conditions Concentration Ranges	Main Results	Reference
Commercial CYN pure standard	Primary rat hepatocytes and KB cells	Incubation with cholate and taurocholate and measurement of CYN uptake across hepatocyte	800 ng/mL for 0, 24, 48, 72 h	There was no protection against the toxicity of CYN at 72 h by both bile acids, although some protection was observed after 48 h. This suggests that bile acid transport may be involve in certain extent in the uptake of the toxin.	[23]
Commercial CYN pure standard	Vero-GFP cells	Monitoring CYN uptake in Vero cells expressing green fluorescent protein (GFP)	0.1–100 µM for 4, 24 h	CYN effects on GFP signal increased 6 fold over 4–24 h incubation indicating slow, progressive uptake of the toxin. However, the mechanism involved was not elucidated.	[32]
Commercial CYN pure standard	Caco-2 cells	Study of intestinal permeability of CYN	1–10 µM for 3, 10, 24 h	The CYN uptake across Caco-2 cells is limited. Only 2.4–2.7% of CYN was detected in the basolateral side after 3 h, increasing slightly up to 16.7–20.5% after 24 h.	[39]
Commercial CYN pure standard	HepG2 cells	Study the influence of cytochrome P450 (CYP) inductors on the cytotoxicity of CYN by means of viability assays	1, 10 µg/L for 4, 12, 24, 48 h	CYPs induction made HepG2 cells more sensitive to CYN toxic effects. Moreover, low concentrations of CYN increased the metabolism in HepG2 cells.	[41]
Commercial CYN pure standard	HepaRG cells and liver tissue fractions	Study of the metabolism of CYN by means of neutral red uptake assay with and without ketaconazol as well as by measuring CYN by LC/MS	0.1–50 µM for 24 h	The use of ketoconazole, a CYP3A4 inhibitor, led to a decreased cytotoxicity of CYN. However, no decrease of CYN was reported after co-incubation with the inhibitor both in HepaRG and liver fractions measured by high resolution mass spectrometry.	[48]
Commercial CYN pure standard	Caco-2 cells	Study of intestinal transport of CYN	0.8 mg/L for 30, 60, 90, 120 min	The paracellular route was pointed out as the most important pathway in CYN absorption. Although a second mechanism was not identified, some insights were reported. This minor carrier-mediated transcellular transport may be independent of energy and Na^+^ and dependent of H^+^ and GSH.	[46]

Abbreviations: Caco-2 (human colorectal adenocarcinoma cell line); cylindrospemopsin (CYN); HepaRG: (human hepatoma cells); HepG2: (human hepatoma cells); KB (human cervix carcinoma); Vero (African green monkey kidney).

**Table 4:** *In vitro* oxidative stress studies dealing with CYN exposure.

Toxin/Cyanobacteria	Experimental Model	Assays Performed	Exposure Conditions Concentration Ranges	Main Results	Reference
Purified extract from *Cylindrospermopsis raciborskii*	Primary rat hepatocytes	GSH	0.8–5 µM CYN for 18 h. Exposure also to CYN + PPG (a GSH synthesis inhibitor)	1.6 µM CYN caused a significant fall (~50%) in cell GSH. At 5 µM GSH cell was only 12.5%. The fall in GSH preceded an increase in LDH release. Reduction of GSH contributes to toxicity. Potentiating effects were found when cells were exposed to CYN and PPG. GSH is most likely to be involved in the detoxification of CYN *in vivo*.	[22]
Purified extract from *Cylindrospermopsis raciborskii*	Primary rat hepatocytes	GSH GSH accumulation GSH efflux GSH synthesis	0, 2.5, 5 µM CYN. Different exposure times depending on the experiment	GSH was depleted significantly after 16 h exposure to 2.5 µM CYN and after 10 h to 5 µM. CYN caused a C-dependent inhibition in GSH accumulation. There was no effect on GSH efflux. GSH synthesis was not altered by 2.5 µM CYN in cell free extracts but the high dilution of the cytosolic content (~500-fold) could avoid the detection of the GSH synthesis inhibition. Authors considered that the inhibition of GSH synthesis is the predominant mechanism for the CYN-induced fall in GSH.	[59]
Purified extract from *Cylindrospermopsis raciborskii* RAC-CYN (synthetic CYN) CYN-DIOL (intermediate from CYN synthesis) EPI-CYN and EPI-DIOL (epimers of CYN at C-7)	Primary rat hepatocytes	GSH	0, 0.16, 0.32, 0.63, 1.25, 2.5 and 5 µM natural CYN and CYN-DIOL 1.25, 2.5, 5, 7.5, 10, 20 µM RAC-CYN	GSH IC_50_ values for CYN and RAC-CYN were 2.38 and 8.99 µM, respectively. When the racemic nature of RAC-CYN and the uncertainty in the original amounts of the synthetic analogues are taken into account, the decrease in GSH by RAC-CYN is almost equivalent to that of natural CYN. CYN-DIOL was as potent as CYN in lowering GSH levels, with the IC_50_ at 2.33 µM. Hepatocytes incubated with 6.25 µM EPI-CYN and EPI-DIOL had cell GSH levels of 39 ± 2.5 and 66 ± 14% of control respectively.	[24]
Purified extract from *Cylindrospermopsis raciborskii*	Primary mouse hepatocytes	GSH LPO by MDA assay	GSH: 0, 1, 5 µM MDA: 5 µM CYN with and without BCNU, an inhibitor of GSSG-Rd	GSH levels were depleted by CYN concentrations of 1 μM and above after a 18-h exposure, and 5 μM produced a significant reduction after 10 h with almost complete depletion after 18 h. However, 5 μM CYN did not elevate levels of lipid peroxidation, as measured by MDA production, and furthermore, inhibition of glutathione reductase by BCNU did not increase MDA production.	[26]
Commercial CYN pure standard	PLCH-1 cells derived from a hepatocellular carcinoma of the topminnow *P. lucida*	ROS GSH GCS activity	0, 2, 4 and 8 mg/mL for 24 h	ROS content increased in a C-dependent way. GSH and GCS activity showed a similar pattern: a significant increase at lowest concentration and a significant reduction at the highest one. The initial increase is considered a try to face the toxic insult. The depletion of GSH may be due to an inhibition of its synthesis.	[34]
Purified extract containing CYN	*Prochilodus lineatus* primary hepatocytes	RONS GST activity G6PDH activity 2GSH/GSSG ratio PCO LPO	0.1, 1.0 or 10 µg/L for 72 h	Cells exposed to the all concentrations of CYN have similar GST and G6PDH activities in comparison to the control group. However, GST activity of the hepatocytes exposed to 10 µg/L was 12% lower than of those exposed to 1 µg/L. G6PDH showed a similar pattern with significant differences between CYN treated cells but not in comparison to the control. No significant alterations were observed for GSH concentration and also for the 2GSH/GSSG ratio. RONS increased 25% in all CYN-exposed groups. PCO did not change. LPO increased in all CYN-exposed groups.	[35]
Commercial CYN pure standard	Human intestinal Caco-2 cell line	ROS GSH GCS activity	0, 0.625, 1.25 and 2.5 µg/mL for 24 h	ROS content was significantly increased only at the concentration of 1.25 mg/mL CYN. GSH and GCS activity were only significantly increased at 2.5 mg/mL. The decrease of ROS at the highest concentration can be related to the higher GSH levels due to its higher synthesis.	[36]
Commercial CYN pure standard	Human vascular endothelium (HUVEC)	ROS GSH GCS activity	0, 0.375, 0.75 and 1.5 µg/mL for 24 h	When HUVEC cells were exposed to 0.375 µg/mL CYN, ROS content was significantly enhanced, while at higher concentrations it decreased to the levels of the control group. GCS activity increased at the highest concentrations (0.75 and 1.5 µg/mL) with enhancements of 2.25 and 3.5-folds, respectively. GSH content underwent concentration-dependent enhancements, with a 3-fold increase at the highest concentration used in comparison with the control group. The recovery of basal ROS content can be related to the concentration-dependent increase in the GSH and the GCS activity observed.	[37]
Commercial CYN pure standard	Human hepatoma cells HepG2	ROS	0.05, 0.1 and 0.5 µg/mL for 5 h	A C-dependent statistically significant increase of ROS was observed in cells treated with 0.05, 0.1 and 0.5 µg/mL CYN already after 30 min of exposure, which steadily increased with incubation time. After 5 h incubation, the fluorescence intensity at the highest dose of CYN was about five times higher than in the control cells.	[60]
Commercial CYN pure standard	Primary rat hepatocytes	ROS Nrf2 transcription factor	0, 90, 180, 360 nM CYN for 24 and 48 h 0, 360 nM CYN with/without 10 or 20 µM resveratrol	CYN induced oxidative stress at all the concentrations tested after 24 and 48 h of incubation. A 3-fold increase in fluorescence was observed in hepatocytes treated with 360 nM CYN for 48 h. Resveratrol partially rescued the cells in a concentration dependent manner after 24 and 48 h of treatment. The increase in cell viability in cultures treated with CYN plus 20 μM resveratrol was about 32% and 7% after 24 and 48 h, respectively, when compared to that of CYN treated cells. A higher level of Nrf2 (transcription factor that regulates the expression of antioxidant enzymes) in toxin treated cells after 48 h was observed.	[38]
Commercial CYN pure standard	Rat hepatic cell line, Clone 9	GSH GCS level	1 µM or 5 µM CYN for 4, 12, 24 and 48 h	Both treatments with CYN (1 and 5 mM) showed a clear and gradual increase of the GSH levels over time, especially at 48 h. No significant changes were observed on GCS level over time in cells exposed to 1 mM. 5 mM CYN, on the contrary, clearly increased levels of GCS time-dependently.	[39]
Commercial CYN pure standard	*Cyprinus carpio* L. leucocyte cell line (CLC)	ROS SOD GSH/GSSG	0, 0.1, 0.5 or 1 μg/mL for 3.5 h	A CYN-induced increase of ROS in exposed CLC cells was observed at each toxin concentration. The results were concentration dependent, with a growing tendency observed until the end of the experiment. In cells exposed to the lowest CYN concentration (0.1 μg/mL) SOD activity was elevated in a statistically significant manner, reaching 179% of the enzyme activity detected in the control cells. At the other tested CYN concentrations SOD activity was also slightly enhanced, however, these increases were not statistically significant. The toxin at each tested concentration increased the total GSH content in the cells, with the concomitant reduction of the GSH/GSSG ratio.	[61]
Purified extract from *Cylindrospermopsis raciborskii*	Human hepatoma cells HepG2	ROS GST activity LPO Superoxide production in mitochondria	0, 0.001, 0.01, 0.1, 1, 10 and 100 µg/L CYN for 48 h with 10% FBS 0, 0.1, 1, 10 µg/L CYN for 12 and 24 h with 2% FBS and/without CYP induction with phenobarbital	No concentration-dependent changes in superoxide production by the mitochondria, ROS and LPO. Actually, LPO decreased. GST activity only increased significantly at 100 µg/L. The 10% FBS could reduced toxicity. ROS increased at both exposure times in an approximate concentration–response pattern, with and without prior CYPs induction. LPO response was very variable; it decreased in non-induced cells exposed to CYN for 12 h and increased in the cells exposed to the highest CYN concentration for 24 h. GST activity only increased after 12 h exposure to 10 µg/L CYN. But on the contrary after 24 h a decreased was observed. CYPs-induction with phenobarbital has led generally to similar results as those observed in non-induced cells for the tested biomarkers.	[41]
Commercial CYN pure standard	Human lymphocytes	ROS SOD activity GPx activity CAT activity LPO	0, 0.01, 0.1 and 1 µg/mL CYN for 0.5–48 h to evaluate ROS production 0, 0.01, 0.1 and 1 µg/mL CYN for 3 and 6 h for the other biomarkers	CYN elevated ROS level in a concentration-dependent manner. The increase was observed within a time as short as 0.5 h of exposure and reached its maximum after 3 and 6 h. SOD level was decreased in a concentration-dependent manner. The greatest depletion (45% respect to the control) was observed after 6 h with 1.0 µg/mL. CAT also decreased after 6 h of exposure to 0.1 and 1 and after 3 h exposure to the highest concentration. GPx activity increased. This was particularly observed after 6 h of exposure. CYN treatments resulted in increased peroxidation of lipids in lymphocytes exposed to 0.1 (after 6 h) and 1 µg/mL (after 3 and 6 h).	[62]
Purified extract from *Cylindrospermopsis raciborskii*	*Hoplias malabaricus* hepatocytes	ROS CAT activity SOD activity GPx activity GST activity G6PDH activity Non-protein thiols GR activity LPO Protein carbonylation	0, 0.1, 1.0, 10, and 100 μg/L for 72 h	The activities of SOD, CAT, GPx, GST and G6PDH were not altered by the exposure to CYN in all groups tested. Non-protein thiols concentration increased 72% only in the cells exposed to the highest CYN concentration. CYN caused a concentration-dependent decrease of GR activity in the cells exposed to >1.0 μg/L. ROS levels increased 40% only in the cells exposed to the highest CYN concentration. No significant damage to lipids (peroxidation), and proteins (carbonylation) was observed.	[63]
CYN Guanidinoacetate (the primary substrate in CYN biosynthesis) 4 CYN synthetic analogs	Human neutrophils	ROS LPO	ROS: 2 µg/mL for 5–60 min LPO: 2 µg/mL for 1 h	All the compounds tested had the ability to temporarily increase the intracellular ROS levels to different extents. LPO levels were significantly increased.	[9]

Abbreviations: BCNU: 1,3-bis(chloroethyl)-l-nitrosourea; CAT: Catalase; CYP: cytochrome P450; G6PDH: Glucose-6-phosphate dehydrogenase; FBS: Fetal Bovine Serum; GCS: Gamma Glutamylcysteine Synthetase; G6PDH: glucose-6-phosphate dehydrogenase; GPx: glutathione peroxidase; GR: Glutathione Reductase; GSH: Glutathione; GST: Glutathione S-transferase; GSSG-Rd: Glutathione disulfide reductase; LPO: Lipid peroxidation; MDA: Malondialdehyde; PCO: Protein carbonylation; PPG: Propargylglycine; RONS: Reactive oxygen/nitrogen species; ROS: Reactive Oxygen Species; SOD: Superoxide dismutase.

**Table 5:** *In vitro* mutagenicity and genotoxicity studies performed with CYN.

Toxin/Cyanobacteria	Experimental Models	Assays Performed	Exposure Conditions Concentration Ranges	Main Results	Reference
Purified extract from freeze-dried *C. raciborskii* culture	Human lymphoblastoid cell line WIL2-NS	Cytokinesis-block micronucleus (CBMN) assay. Micronuclei (MN) were counted in binucleated cells (BNCs)	1,3,6, and 10 µg CYN/mL, 24 h and 48 h	CYN induced significant increases in the frequency of MN in BNCs exposed to 6 and 10 µg/mL, and a significant increase in centromere (CEN)-positive MN at all concentrations tested. At the higher concentrations, both CEN-positive and CEN-negative MI were induced.	[72]
Purified extract from a *Cylindrospermopsis raciborskii* Australia strain (AWQC CYP-026J)	Chinese hamster ovary K1 (CHO-K1) cells	Comet assay	0.5–1.0 µg CYN/mL, 24 h	No significant induction of DNA strand breaks could be detected after 24 h treatment. However, cell growth was inhibited, as well as cell blebbing and rounding.	[73]
Purified extract from *Cylindrospermopsis raciborskii*	Primary mouse hepatocytes	Comet assay	0.05–0.5 µM CYN. Moreover, cells were preincubated with inhibitors of CYP450: SKF525A, omeprazole	CYN induced increases in comet tail length, area and tail moment at 0.05 µM. The CYP450 inhibitors completely inhibited the genotoxicity of CYN.	[26]
Purified extract from *C. raciborskii* (AWT205)	Human dermal fibroblasts (HDFs), Caco-2, HepG2 and C3A cells	Quantification of mRNA levels for selected p53-regulated genes using qRT-PCR	1, 2.5, or 5 µg/mL CYN for 6 h or 24 h	After 6 h exposure to CYN, concentration-dependent increases in mRNA levels were observed for the p53 target genes *CDKN1A*, *GADD45α*, *BAX* and *MDM2*, indicating an early activation of p53, which remained elevated after 24 h of exposure.	[27]
Purified extract from two cultures of *C. raciborskii* (AWT 205, and CYN-Thai)	CHO-K1 cells	Chromosome aberration (CA) assay	0.05–2 µg CYN/mL were assayed. DNA damage was determined after 3, 16 and 21 h of exposure and the assay was performed with and without metabolic activation (S9)	CYN with and without S9 had no significant influence on the frequency of CA.	[28]
Commercial pure standard CYN (>98% purity)	Caco-2 and HepaRG cells, differentiated and undifferentiated cells	Cytokinesis-block micronucleus (CBMN) assay	0.5–2 µg CYN/mL, and the CYP450 inhibitor ketoconazole (1–5 µM) for 34 h	CYN increased the frequency of binucleated cells in both cell lines, and ketokonazole reduced both the genotoxicity and cytotoxicity induced by CYN	[53]
Purified extract containing CYN	Hepatocytes of the fish *Prochilodus lineatus*	Comet assay	0.1, 1.0, or 10 µg CYN/L for 72 h	No significant effects on DNA strand breaks were found.	[35]
Commercial pure standard CYN	HepG2 cell line (human hepatoma cell line)	Comet assay, and MN, nuclear bud (NBUD), nucleoplasmic bridge (NPB) formation. Changes in the expression of genes involved in the response to DNA damage and in CYN metabolism were investigated using real-time quantitative PCR (qPCR)	0–0.5 µg CYN/mL) for 4, 12, and 24 h	Non cytotoxic concentrations of CYN (0–0.5 µg/mL) induced increased DNA strand breaks after 12 and 24 h of exposure. Increased frequency of MN, NBUDs and NPBs after 24 h exposure in a dose-dependent manner was reported. CYN upregulated the expression of the *CYP1A1*, *CYP1A2* genes, and the expression of the P53 downstream-regulated genes *CDKN1A*, *GADD45α*, and *MDM2*.	[74]
Commercial pure standard CYN	Human peripheral blood lymphocytes (HPBLs)	Comet assay and the cytokinesis-block micronucleus (CBMN) assay. Gene expression of *CYP1A1*, *CYP1A2*, *P53*, *MDM2*, *GAdd45α*, *CDKN1A*, *BAX*, *BCL-2*, *GCLC*, *GPX1*, *GSR*, *SOD1* and *CAT*, using the qPCR	The whole blood was treated with CYN (0, 0.05, 0.1 and 0.5 μg/mL) for the comet and CBMN assays. For the mRNA expression the isolated HPBLs were exposed to 0.5 μg/mL of CYN for 4 and 24 h	In HPBLs CYN induced the formation of DNA single strand breaks (comet assay), a time and dose-dependent increase in the frequency of MN and NBUD was observed, and a slight increase in the number of NPB. CYN up-regulated the genes *CYP1A1* and *CYP 1A2*, and the mRNA expression of some DNA damage (*P53*, *GADD45α*, *MDM2*), apoptosis responsive genes (*BAX*, *BCL-2*), and some genes involved in the antioxidant enzymes (*GPX*, *GSR*, *GCLC*, *SOD1)* whereas no changes were detected in *CDKN1A* and *CAT*.	[75]
Commercial pure standard CYN and crude extracts from cyanobacterial blooms. Mixture of commercial pure toxins: CYN, MC-LR and anatoxin-a	*Salmonella typhimuriun* strains (TA 98, TA 100, TA1535, TA 1537) and *Escherichia coli* WP2 uvrA and WP2 (pKM101)	Mutagenicity: Ames test	Pure CYN: 0.312, 0.625, 1.25, 2.5, 5 and 10 µg/mL. The mixture of pure toxins (CYN, MC-LR and anatoxin-a) at 1 µg/mL was also tested but only in two *Salmonella typhimuriun* strains (TA 98, TA 100)	Mutagenicity was detected in four of the ten extracts assayed, mainly against *S. typhimurium* TA100. By contrast, pure CYN was not mutagenic towards all the six bacterial strains up to a concentration of 10 µg/mL. No effects were detected after bacteria exposure to the mixture of purified toxins.	[76]
Commercial CYN Pure standard	Common carp (*Cyprinus carpio*) leukocytes	Alkaline version of comet assay	0.5 µg CYN/mL for 18 h	The cells treated with CYN were affected to a lesser extent in comparison to the damage induced by MC-LR.	[77]
Commercial CYN Pure standard	HepG2 cell line (human hepatoma cell line)	Formation of double strand breaks (DSBs). Analysis of the cell-cycle by flow-cytometry	0–0.5 µg CYN/mL for 24–96 h	CYN induced formation of DSBs after 72 h exposure. The toxin has impacts on the cell cycle, indicating G0/G1 arrest after 24 h and S-phase arrest after longer exposure (72 and 96 h).	[78]
Commercial CYN Pure standard	HepG2 cells	Gene expression was analyzed by qPCR	0.5 µg CYN/mL for 12 and 24 h	CYN increased expression of the immediate early response genes, and strong up-regulation of the growth arrest and DNA damage inducible genes (*GADD45α, GADD45β*), and genes involved in DNA damage repair (*XPC, ERCC4* and others). Up-regulation of metabolic enzyme genes provided evidence for the involvement of phase I and phase II enzymes in the detoxification response and potential activation of CYN.	[79]
Commercial CYN Pure standard	HepG2 cells	Alkaline comet assay and Fpg -enzyme modified assay	0–0.5 µg CYN/mL) for 4, 12 and 24 h	No DNA damage was observed after 4 h exposure to CYN. After 12 and 24 h, CYN (0.25–0.50 µg/mL) induced significant increase of DNA strand breaks, but not oxidative damage. CYN did not induce apoptosis.	[60]
Treated water; crude extract of *C. raciborskii* (CYP-011K); crude extract containing CYN; no toxic extract	HepG2 cells	Comet assay	Cells were exposed to all extracts at concentration of 0.1, 0.5 and 1 µg of dry material/mL, and also to treated water only, for 24, 48 and 72 h	DNA damage was detected only under toxic *C. raciborskii* extract, at the concentration of 1 µg/mL from 24 h of exposure, and at 0.5 µg/mL after 48 and 72 h.	[80]
Commercial CYN Pure standard	HepG2 cells with a plasmid that encodes the fluorescent protein DsRed2 under the control of the *CDKN1A* promoter, (HepG2CD-KN1A-DsRed cells)	The induction of the DsRed fluorescence intensity was determined by spectrofluorimetry, fluorescence microscopy and flow cytometry	Cells were exposed to CYN and the DsRed fluorescence was determined at 24 and 48 h of exposure; the cell viability was determined at 48 h	LOEC ^2^: 0.12 μM and RDF ^3^: 1.53 μM	[81]
Commercial CYN Pure standard	*Cyprinus carpio* L. leucocyte cell line (CLC)	The cytokinesis-block micronucleus (CBMN) assay. The fluorimetric OxyDNA assay kit was also employed	0.1, 0.5, or 1 µg CYN/mL, for 24 h	CYN increase the number of MN, and oxidative DNA damage was also detected.	[61]
Commercial CYN and MC-LR pure standards, and mixtures MC-LR/CYN	HepG2 cells	Alkaline comet and CBMN assays were performed. The expression of selected genes was analyzed by quantitative time PCR	CYN: 0.01–0.05 µg CYN/mL; MC-LR: 1 µg/mL, and MC-LR/CYN mixtures for 4 h and 24 h	CYN after 24 of exposure induced DNA stand breaks and genomic instability. The MCLR/CYN mixture induced DNA strand breaks after 24 h exposure, but to a lesser extent as CYN alone. The induction of genomic instability and changes in the expression of selected genes induced by the mixture were similar to those induced by CYN alone. CYN alone resulted in changes in the expression of genes involved in the metabolism (*CYP1A1*, *CYP1A2*, *NAT2*), genes involved in immediate-early response/signaling (*FOS*, *JUN*, *TGFB2*), and DNA damage (*MDM2*, *CDJN1A*, *GADD45A*, *ERCC4*), while MC-LR alone down-regulated the expression of *NAT2* and *TGFB2*. The binary mixture exhibit similar results that CYN alone.	[82]
Purifies extract from the strain *C. raciborskii* CYPP011K	*Hoplias malabaricus* hepatocytes	Comet assay	0.1–100 µg/L of CYN for 72 h	No significant DNA damage was observed	[63]

^1^ Fpg: Formamidopyrimidine glycosylase enzyme; ^2^ LOEC: Lowest effective concentration that induced ≥1.5-fold increase in relative DsRed fluorescence, over the solvent-treated control; ^3^ RDF: Relative DsRed fluorescence induction detected at LOEC.
